# Case Report: The first case of successful pregnancy and live birth following laparoscopic resection of adenomyosis under real-time intraoperative ultrasound elastography guidance

**DOI:** 10.3389/fmed.2024.1457611

**Published:** 2024-09-26

**Authors:** Yoshiaki Ota, Kuniaki Ota, Toshifumi Takahashi, Naoki Horikawa, Ryosuke Kuroda, Hana Okamoto, Yu Tanaka, Tomoyuki Kusumoto, Takashi Oda, Takehiko Matsuyama, Takahito Miyake, Tetsuro Honda, Koichiro Shimoya

**Affiliations:** ^1^Department of Gynecologic Oncology, Kawasaki Medical School, Okayama, Japan; ^2^Fukushima Medical Center for Children and Women, Fukushima Medical University, Fukushima, Japan; ^3^Department of Obstetrics and Gynecology, Kurashiki Chuo Hospital, Okayama, Japan; ^4^Department of Obstetrics and Gynecology, Miyake Clinic, Okayama, Japan; ^5^Department of Obstetrics and Gynecology, Koujin Hospital, Kagawa, Japan

**Keywords:** adenomyosis, infertility, laparoscopic adenomyomectomy, intraoperative elastography, uterine rupture, embryo transfer, placenta accreta spectrum, twin pregnancy

## Abstract

**Objective:**

Adenomyosis-related infertility is increasingly being diagnosed, and surgical intervention has been suggested to improve fertility. Elastography, a noninvasive ultrasound technique, is promising for diagnosing and guiding the resection of adenomyosis. This report presents the first case of successful delivery after twin pregnancies achieved with IVF following intraoperative elastography-guided laparoscopic adenomyomectomy.

**Case report:**

A 35-year-old Japanese woman with uterine adenomyosis received a gonadotropin analog before surgery. Preoperative MRI revealed a 5.0 × 7.0 cm adenomyoma, leading to scheduled laparoscopic adenomyomectomy with intraoperative elastography. During surgery, elastography ensured the complete resection of the adenomyotic tissue while preserving the endometrium. Postoperative MRI confirmed the absence of residual adenomyosis. The patient underwent *in vitro* fertilization and embryo transfer, leading to a successful twin pregnancy after double blastocyst transfer. Despite a stable perinatal course, she required hospitalization to prevent preterm labor. At 32 weeks, an elective cesarean section delivered healthy twins. The intra- and post-operation was uncomplicated, and the patient and infants had an optimal health.

**Conclusion:**

This is the first reported case of a twin pregnancy resulting from vitrified-warmed embryo transfer after elastography-guided laparoscopic adenomyomectomy, culminating in a successful delivery via cesarean section. This technique allows precise resection and mitigates the risks of uterine rupture and placenta accreta spectrum disorders. Although promising, further studies are required to validate the safety and efficacy of this innovative surgical approach.

## 1 Introduction

Patients with infertility are increasingly diagnosed with adenomyosis (1, 2). The mechanisms of infertility in patients with adenomyosis include intrauterine abnormalities such as adenomyomas, which May obstruct the tubes and distort the uterine cavity (2, 3). However, emerging evidence suggests that surgical intervention improves fertility (4–8). Focal adenomyosis refers to localized adenomyotic lesions or nodules, while diffuse adenomyosis involves more widespread involvement of the myometrium (8, 9). Hysterosalpingography studies have shown uterine cavity distortion in 78% of patients with diffuse adenomyosis compared to 54% with focal adenomyosis (10). Therefore, diffuse adenomyosis uteri requires aggressive infertility treatment such as the surgical intervention because the impact on fertility May differ, with diffuse adenomyosis potentially having more widespread effects on uterine function. Concretely, diffuse adenomyosis uteri tends to be treated with laparotomy or laparoscopy because the adenomyotic lesion cannot be directly palpated (11, 12). Laparoscopic adenomyomectomy is associated with the risk of too little or extensive resection since the boundary between the adenomyoma and myometrium was ill-defined, resulting in upregulating recurrence rates of adenomyosis or the rate of uterine rupture depending on the extent of excision (13, 14).

Elastography, a medical imaging procedure that is capable of measuring tissue mechanical properties and elasticity, is expected to be a noninvasive diagnostic ultrasound for uterine adenomyosis (15). A recent meta-analysis found that elastography had high sensitivity and specificity for adenomyosis diagnosis, and its findings correlated with histopathological results (16). In 2020, we developed a surgical technique that allows complete resection of adenomyotic lesions using intraoperative elastography (17). To date, only a few have reported that laparoscopic excision of an adenomyotic lesion using intraoperative ultrasound elastography is a feasible technique to completely resect adenomyotic lesions (17–19).

We report the first case of successful twin delivery after two vitrified-warmed embryo transfers and intraoperative elastography-guided laparoscopic adenomyomectomy. Two neonates were delivered by elective cesarean section at 32 weeks following perinatal management.

## 2 Case presentation

A 35-year-old nulliparous Japanese woman had no past medical history or family history, had suffered from dysmenorrhea for several years, and was diagnosed with transvaginal ultrasound as having uterine adenomyosis at the previous gynecology outpatient clinic. She received gonadotropin analog agonist (leuprorelin acetate®, 1.88 mg, ASUKA Pharmaceutical Co., Ltd) for 6 months and following continuously taking dienogest (Dinagest tab®, 2 mg/day Mochida Pharmaceutical Co., Ltd.) with the same protocol (20). The medications were discontinued after the patient decided to conceive. After that, she underwent the program for *in vitro* fertilization (IVF) and intracytoplasmic sperm injection (ICSI) because she did not conceive after three ovulation induction cycles combined with intrauterine insemination. After adequate hormonal stimulation of ovarian follicles, she had transvaginal oocyte retrieval of 6 mature oocytes and underwent conventional IVF or ICSI resulting in preserving 4 high-quality frozen blastocysts as evaluated using Gardner’s classification (21). She underwent three times of vitrified-warmed blastocyst transfers using hormone replacement therapy as previously described (22), and all transfers failed unfortunately. Therefore, the reproductive medicine specialist advised her to treat adenomyosis as the causal factor of implantation failure, she visited our department for uterine adenomyosis surgery. Thereafter, the reproductive medicine specialist and we discussed the therapeutic strategy, and she decided to undergo laparoscopic adenomyomectomy with intraoperative elastography initially, and subsequently retrieve oocytes in the IVF program to transfer embryos following completely healing the myometrial wounded after the enucleation.

**Figure 1:**
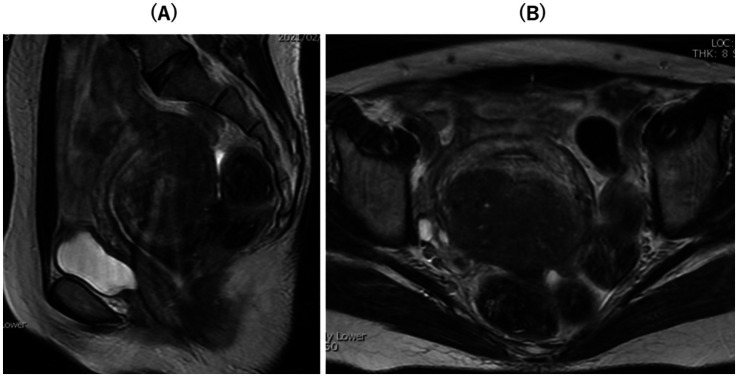
The preoperative T2-weighted magnetic resonance imaging (MRI). **(A)** Sagittal T2-weighted preoperative pelvic MRI showed diffuse adenomyosis with thickening of the junctional zone in the posterior wall and severe adhesion on the pouch of Douglas. **(B)** Axial T2-weighted postoperative pelvic MRI showed intramural solid adenomyosis.

**Figure 2:**
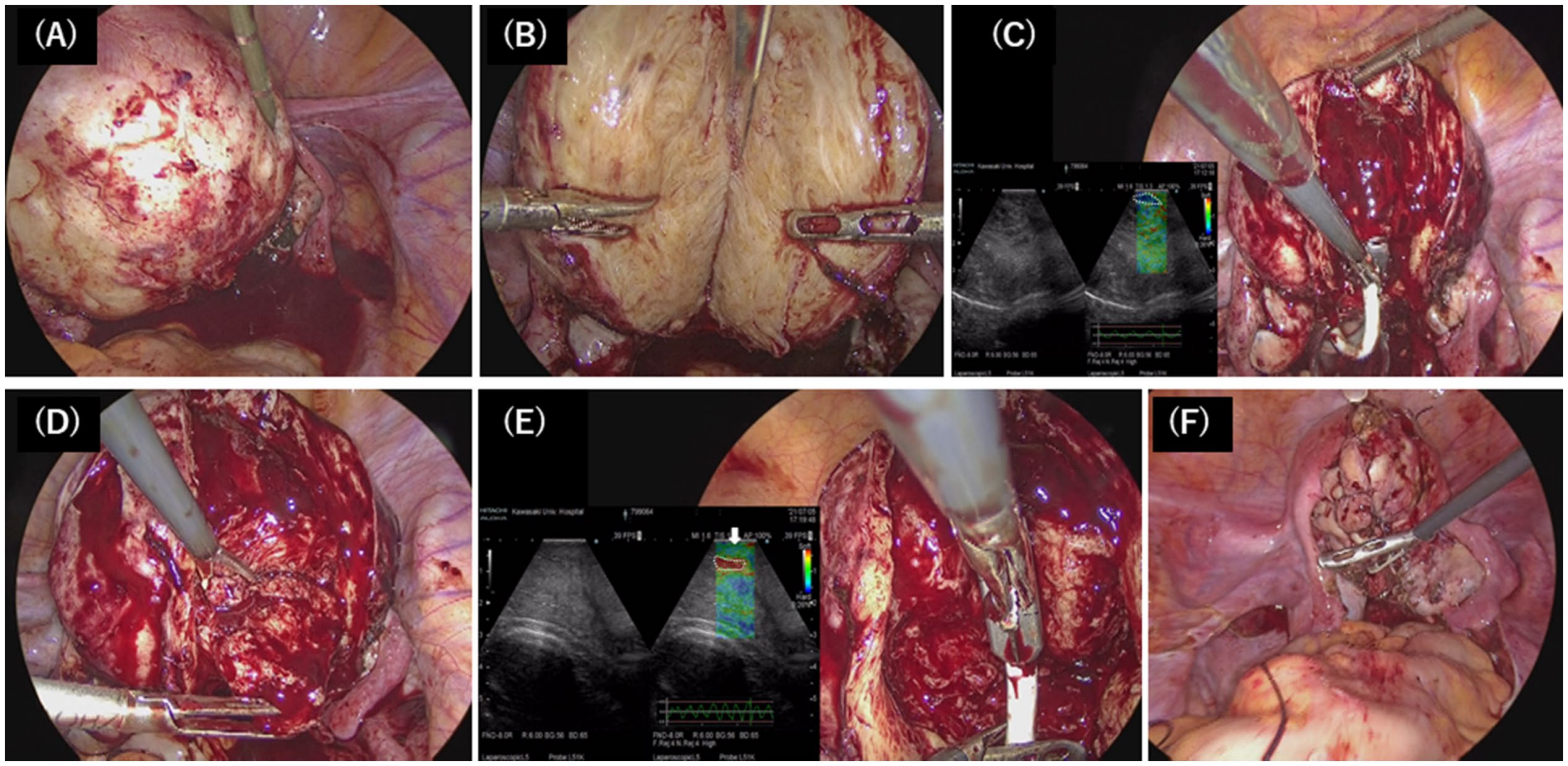
Surgical technique of laparoscopic adenomyomectomy. **(A)** Vasopressin was injected into the uterine wall. **(B)** The uterus was longitudinally incised with the scalpel to directly access the adenomyotic tissue, and the nucleation was resected in a wide wedge shape. **(C)** The probe of the elastography was applied on the dissected uterine wall to detect the residual adenomyotic tissue, indicated by the bright blue areas (white-dotted circle). **(D)** The residual adenomyotic tissues were resected by scissors forceps. **(E)** After complete resection, the bright blue areas were changed to bright green areas, which indicated the normal layer of the myometrium (white arrow), and the endometrium was indicated by the bright red areas (white dotted circle). **(F)** The uterine incisions were repaired in multiple layers using barbed sutures.

**Figure 3:**
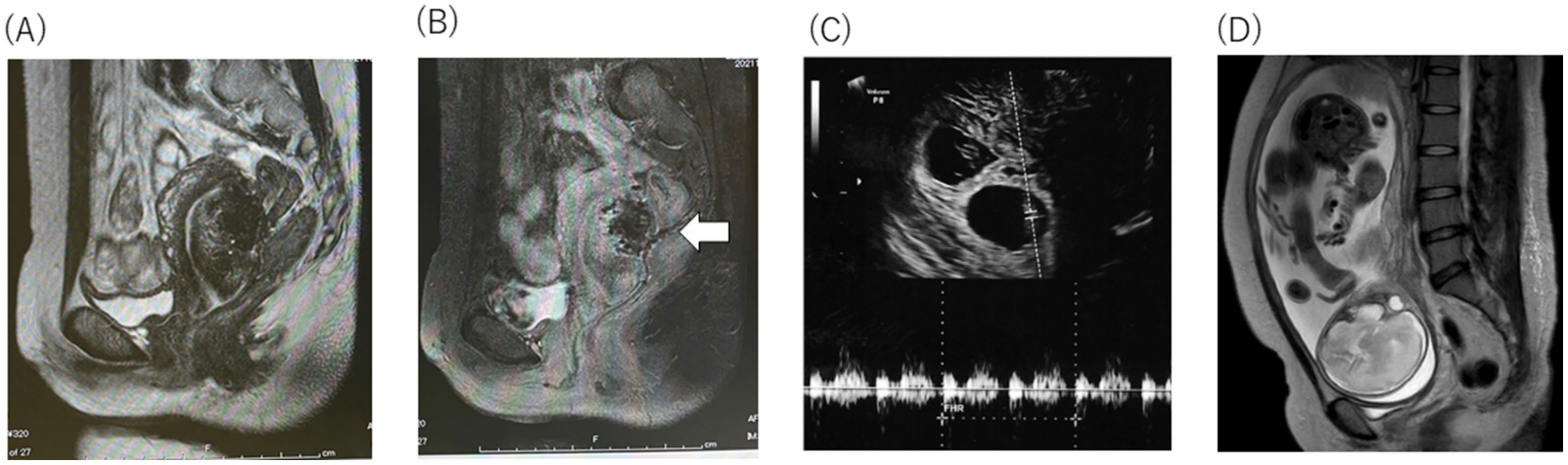
The preoperative T2-weighted magnetic resonance imaging (MRI). **(A)** Sagittal T2-weighted postoperative pelvic MRI showed o adenomyotic lesion and the complete resection macro-imagingly. **(B)** Sagittal T2-weighted postoperative contrast-enhanced pelvic MRI showed incomplete blood flow in the posterior wall of the uterine (white arrow), and the normal repair process of the myometrium. **(C)** Two embryos with heartbeats at 8 weeks 0 day were observed with M-mode (white dotted line) **(D)** T2-weighted MRI revealed that the uterine wall remained circumferentially thick.

Preoperative T2-weighted magnetic resonance imaging (MRI) mainly showed diffuse thickening of the junctional zone in the posterior wall, leading to the diagnosis of a 5.0 × 7.0 cm intramural solid adenomyoma (Figures 1A,B). Therefore, we scheduled a laparoscopic adenomyomectomy with intraoperative elastography for a more accurate resection. This technique has been previously described (17, 18). Briefly, the intraoperative course of this case combined with this surgical technique was described below. Laparoscopy revealed a 12-week gestation-sized enlarged uterus with obliteration of the pouch of Douglas due to endometriosis; adhesions were promptly dissected. Vasopressin (1 IU/70 mL normal saline) was injected into the uterine wall to minimize bleeding (Figure 2A). A no. Eleven scalpel was introduced into the abdomen after removing the trocar, and a longitudinal incision was made over the uterine fundus (Figure 2B). Adenomyotic lesions in the posterior uterine wall were meticulously excised. After initial enucleation, a laparoscopic ultrasound probe was used to detect any remaining tissue. A posterior 1–2 cm transverse colpotomy was performed in the midline of the posterior fornix, marked with a Vagi-Pipe® (Hakko Medical, Nagano, Japan). An ultrasound probe (ARIETTA 850, Hitachi, Ltd., Tokyo, Japan) inserted through the colpotomy identified the residual adenomyosis as bright blue areas (Figure 2C). Tissues were resected using scissor forceps (Figure 2D). Subsequent real-time elastography confirmed the complete removal of the adenomyosis, preserving the endometrium (Figure 2E). The resected specimens were extracted via colpotomy. The uterine incisions were repaired in multiple layers using barbed sutures (0 Stratafix® Symmetric PDS® Plus, Ethicon Endo-Surgery, Tokyo, Japan) (Figure 2F). After myometrium reapproximation and hemostasis, an adhesion barrier (Interseed®, Ethicon Endo-Surgery, Tokyo, Japan) was placed. The surgery was completed without complications and lasted 188 min, with an estimated blood loss of 100 mL (Supplementary Video S1). The resected lesion weighed 102.5 grams. The patient’s postoperative recovery was uneventful. A 12-week postoperative MRI showed sufficient myometrial thickness and no residual adenomyotic lesions in the resected area (Figures 3A,B).

**Figure 4:**
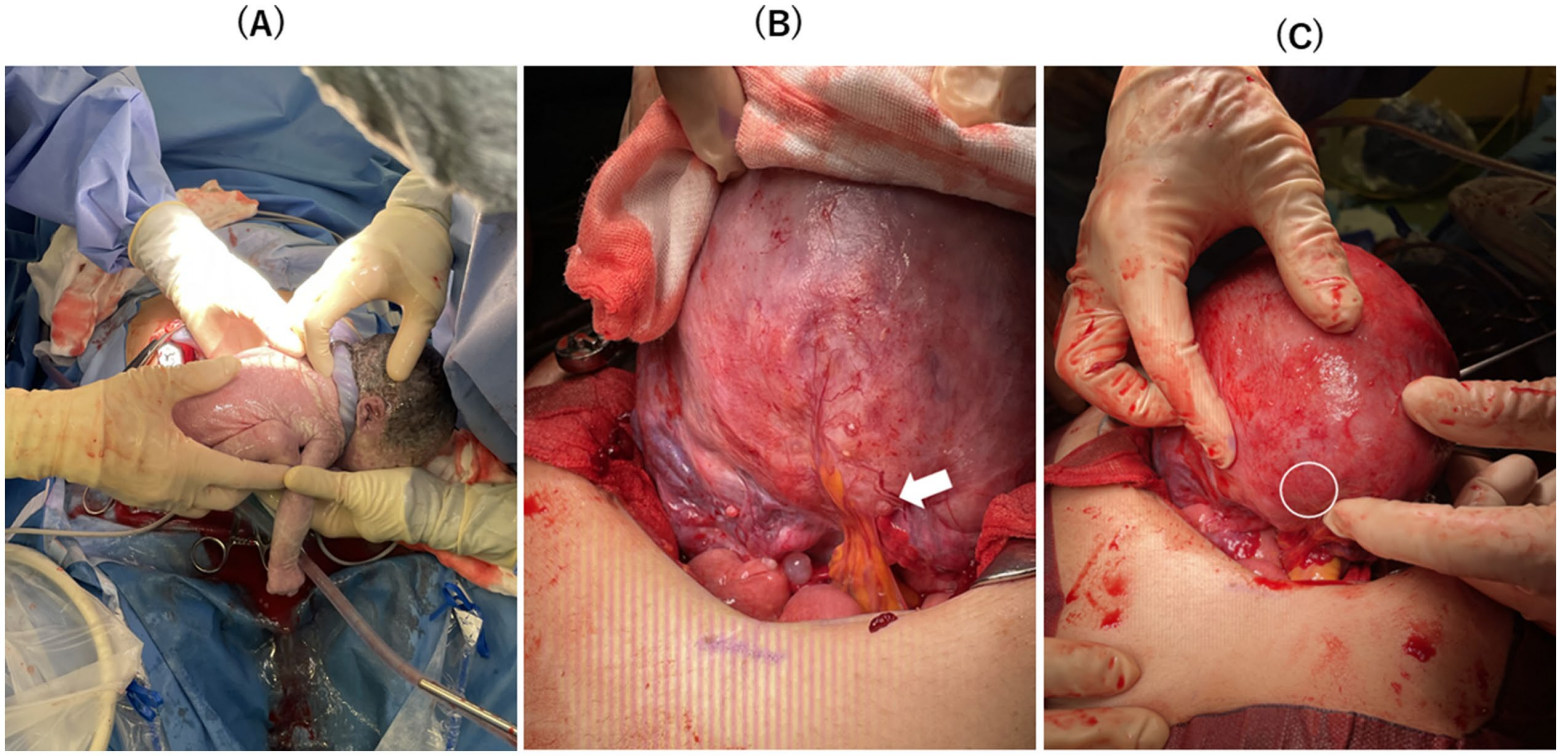
**(A)** Healthy neonates were delivered. **(B)** There was one adhesion between the lower margin of the posterior uterine wall wound and mesenteric fat at the pouch of Douglas (white arrow). **(C)** The thumb-sized thin myometrium on the posterior wall was slightly confirmed (white circle), although there was no sign of uterine rupture.

Five months after surgery, she began an *in vitro* fertilization and embryo transfer (IVF-ET) program and successfully preserved multiple high-quality frozen blastocysts as evaluated using Gardner’s classification (21). She underwent vitrified-warmed blastocyst transfer using hormone replacement therapy with same protocol of hormone replacement therapy (22). After three failed attempts with single blastocyst transfers, she consented to a double blastocyst transfer due to recurrent implantation failures, was informed of the risks, such as placenta accreta spectrum (PAS) and uterine rupture, and obtained permission for maternal–fetal invasive care unit (MFICU) monitoring. At 5 weeks, transvaginal ultrasonography revealed a dichorionic diamniotic twin pregnancy, with gestational sacs measuring 9.4 and 8.3 mm. By 8 weeks, the crown-rump lengths were 15.7 mm and 13.8 mm, with detectable fetal heartbeats (Figure 3C). Despite a stable perinatal course at 26 weeks, she was admitted for magnesium sulfate infusion to prevent preterm labor due to a short cervical canal (5 mm) with funneling membranes and uterine contractions. This treatment was effective and no further preterm labor was observed. At 27 weeks, MRI showed no placenta accreta or significant thinning of the muscle layer because the placenta was on the anterior wall (Figure 3D). An elective cesarean section was scheduled at 32 weeks to prevent uterine rupture, and betamethasone (12 mg every 24 h, 2 doses) was preoperatively treated to promote the maturity of the fetal lung. The procedure at 32 weeks and 0 days resulted in the delivery of healthy twins: one weighing 1,650 g with Apgar scores of 8 (1 min)/ 9 (5 min) and 7.346 of umbilical artery pH, and the other weighing 1,466 g with Apgar scores of 4 (1 min)/ 7 (5 min) and 7.244 of umbilical artery pH (Figure 4A). There were postnatal abnormalities such as respiratory distress syndrome or intraventricular hemorrhage in both neonates. The placenta was removed manually without complications. Upon exteriorizing the uterus for myometrial suturing, adhesions to the posterior uterine wall and a thin myometrium were confirmed (Figures 4B,C). The operation lasted 87 min, with an estimated blood loss of 2,164 mL. The patient recovered without complications and was discharged on postoperative day five. The infants were discharged on day 28 in excellent condition, requiring no oxygen or feeding tubes.

## 3 Discussion

Here, we reported the first case of a twin pregnancy conceived through vitrified-warmed embryo transfer after intraoperative elastography-guided laparoscopic adenomyomectomy, resulting in the delivery of two neonates by elective cesarean section at 32 weeks with careful perinatal management. Despite the high risk of uterine rupture and PAS after adenomyomectomy, this novel fertility-sparing technique using intraoperative elastography enabled successful live births.

The prevalence of uterine rupture during pregnancy following adenomyomectomy is reported to be 2.8–12.5% (23–25). The risk of uterine rupture in twin pregnancies after laparoscopic adenomyomectomy is unknown, with only three reported cases (26–28). The first case reported that spontaneous uterine rupture at 30 weeks of gestation occurred after a monochorionic twin pregnancy following IVF-ET 12 months post-surgery (26). The second case reported successful delivery at 31 weeks of gestation with an elective cesarean section for a monochorionic twin pregnancy 30 months after IVF-ET (27). The third case reported that the uterine was already ruptured and two babies expired before the patient was emergently transferred to the hospital at 30 weeks of gestation (28). In particular, Kweon et al. reported a case–control study of five cases of twins after adenomyomectomy, all of which resulted in emergency cesarean section due to preterm delivery, but the most successful pregnancy course was at 33 weeks of gestation, and the earliest stage was at 28 weeks of gestation (28). To our best knowledge, there have been no cases of successful planned delivery after 34 weeks of gestation in twins after adenomyomectomy. Hence, we consulted with pediatricians in MFICU to decide on plan delivery at least at 32 weeks of gestation, with mature fetal lungs by predelivery betamethasone, thus avoiding prematurity and related complications of perinatal morbidity and mortality. Consequently, live birth was achieved at 32 weeks without uterine rupture, despite a slight myometrial defect undetected by perinatal ultrasound.

Several reports have been published regarding the prevention and prediction of uterine rupture after adenomyosis enucleation. Some studies recommend using a cold scalpel instead of electrocautery during uterine incision to prevent rupture (29–31). Additionally, electrosurgery and multilayered myometrial closures should be avoided because they May predispose the wound to rupture owing to poor healing characteristics (30, 32). Otsubo et al. examined uterine wall thickness in patients undergoing abdominal adenomyomectomy for diffuse adenomyosis and found that a uterine wall thickness of less than 7 mm increased the risk of uterine rupture (33). Kwak et al. recommended regular monitoring of uterine contractions and wall thickness to prevent rupture (27). In this case, postoperative and prenatal MRIs confirmed myometrial preservation, and the patient successfully delivered at 32 weeks without rupture; however, we attributed our success to our adherence to previously reported methods of preventing uterine rupture.

Patients with a history of uterine surgery, including adenomyomectomy, have a higher likelihood of preterm delivery. After undergoing adenomyomectomy, patients have shorter cervical lengths upon admission and a significantly higher rate of cervical length shortening (34). Extensive myometrial removal, particularly from the caudal uterus, May have contributed to this issue. Kwak et al. performed an elective cesarean section at 31 weeks in a twin pregnancy after laparoscopic adenomyomectomy for cervical shortening (27). Given these risks, we recommend scheduling an elective cesarean section at an appropriate time in consultation with a pediatrician and the MFICU rather than waiting until term, although the exact mechanism of uterine contraction and cervical shortening post-adenomyomectomy remains unclear.

Another serious pregnancy complication after adenomyomectomy is PAS, which can lead to uterine rupture. Recently, Sayama et al. conducted a retrospective study to evaluate the impact of adenomyomectomy on pregnancy outcomes in women with adenomyosis. The results revealed that while adenomyomectomy reduced the incidence of complications such as preterm prelabor rupture of membranes, preeclampsia, and small-for-gestational-age infants, it also increased the risk of PAS (35). Embryo implants at the myometrial incision site can cause PAS and rupture during pregnancy. Sumigawa et al. noted that the incidence of PAS after uterine surgery May depend on the suture method used for myometrial closure (36). In this case, we speculate that the placement outside the adenomyomectomy site and the use of barbed sutures for closure May have prevented PAS. Further studies are required to evaluate whether surgical techniques using barbed sutures reduce the incidence of PAS. However, it is also possible that our technique is uniquely superior because elastography can easily depict the hardness and softness of the tissue and detect the endometrium indicated by the bright red areas. Areas (Figure 2E), although Wada et al. concluded that one of the risks of PAS is intraoperative uterine cavity breach (37). Therefore, perforation of the uterine cavity May be avoided with elastography.

Adenomyotic lesions May disturb the process of spiral artery remodeling in the myometrial junctional zone from the onset of decidualization, causing defective placentation (38, 39), and consequently, the risk of fetal growth restriction (FGR) May increase due to placental blood flow during pregnancy (40).In retrospective cohort study, Ono et al. concluded that the laparoscopic adenomyomectomy had a significantly lower prevalence of FGR compared to the no surgery group (41). In this case, we did not diagnose FGR, albeit at the lower end of the normal range of fetal weight due to twin, and we speculated that elastography ensured resection of the adenomyosis foci, which promoted normal placentation.

In conclusion, to the best of our knowledge, this is the first report of dichorionic twin delivery without uterine rupture after laparoscopic adenomyomectomy under elastography guidance. Adenomyomectomy is a uterus-preserving surgical treatment for adenomyosis that offers symptom relief and potential fertility preservation but comes with risks of recurrence, surgical complications, and potential uterine rupture in future pregnancies (42). Hence, the success of adenomyomectomy heavily depends on surgical expertise, with experienced surgeons better equipped to handle the procedure’s complexity, select appropriate cases, manage complications, and implement evolving techniques (43). While adenomyomectomy can be effective if the selection of case was correct, its use requires careful consideration of individual patient factors, and ongoing research is needed to optimize long-term outcomes and refine patient selection criteria. On the other hand, Elastography, particularly shear wave elastography, has emerged as a valuable tool for improving diagnosis, surgical planning, and treatment monitoring. This technique May have the possibility to allow precise resection and mitigate the risks of uterine rupture and PAS, although adenomyomectomy is not strongly recommended, and meticulous surgical techniques are important to minimize uterine defects. The use of elastography in this context lacks robust evidence and is currently evolving through clinical research. Further studies involving larger cohorts are required to establish the safety and efficacy of laparoscopic elastography-guided adenomyomectomies.

## Data Availability

The original contributions presented in the study are included in the article/Supplementary material, further inquiries can be directed to the corresponding author.
